# Japanese Legacy Cohorts: Six-Prefecture Cohort Study (Hirayama Cohort Study)

**DOI:** 10.2188/jea.JE20190249

**Published:** 2020-03-05

**Authors:** Suminori Akiba, Yoshihide Kinjo

**Affiliations:** 1 Specially Appointed Professor, Hirosaki University, Aomori, Japan; 2 Professor Emeritus, Kagoshima University, Kagoshima, Japan; 3 Okinawa Prefectural College of Nursing Graduate Study in Health Nursing, Naha, Japan

**Keywords:** cohort study, smoking, cancer epidemiology

## Abstract

Late Dr Takeshi Hirayama and his colleagues conducted a mortality follow-up of a large-scale cohort in six prefectures in Japan. This study is called the six-prefecture cohort study or Hirayama Cohort Study. The study subjects were residents aged 40 years or older at the baseline survey in 1965, which covered 94.8% of residents identified in the study area by the National Census conducted on October 1, 1965. The mortality of 264,118 cohort members was followed until the end of 1982. One of the most important findings made by this study was an association between second-hand smoke exposure and lung cancer. This finding is the origin of the worldwide spread of smoking ban in indoor public venues and workplaces. Other major findings obtained from the study are also briefly described in this article.

## ORIGIN OF THE COHORT

**Figure 1.**
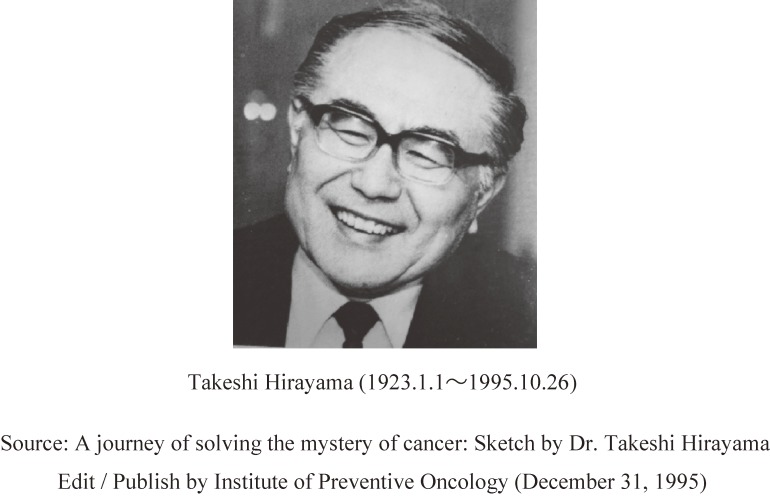


After conducting more than 30 case-control studies in Japan during the period between 1952 and 1964, late Dr Takeshi Hirayama (Figure 1) felt it necessary to take another epidemiological approach.^1^ Inspired by large-scale cohort studies^2^^–^^6^ conducted in North America and the United Kingdom in the 1950s, Dr Hirayama decided to start the six-prefecture cohort study (Hirayama Cohort Study) in Japan in order to examine the cause-specific mortality in relation to lifestyles such as cigarette smoking, alcohol drinking and dietary habits.^1^^,^^7^ Main features of Hirayama Cohort Study were that it adopted a census population-based approach and targeted a wide range of lifestyles: tobacco smoking, alcohol drinking, dietary intake, and typical lifestyles in Japan such as Japanese tea drinking.

## BUILDING AND MAINTAINING THE COHORT

### Study populations and areas

**Figure 2.**
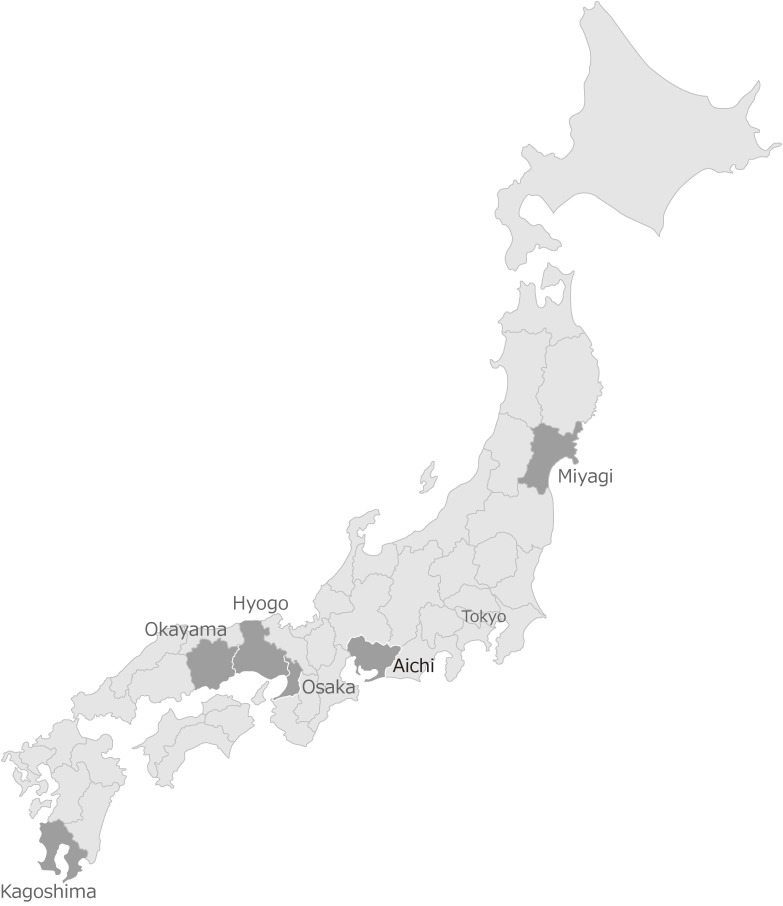
Geographical location of the prefectures sampled in Japan.

From October to December 1965, a questionnaire survey (the baseline survey) of all residents aged 40 or older was conducted in 29 public health districts in six prefectures (Miyagi, Aichi, Osaka, Hyogo, Okayama and Kagoshima). Those prefectures (Figure 2), selected by Dr Hirayama, geographically represent the entire Honshu Island, one of five major islands of the archipelago of Japan.^7^ From 7 prefectures in Kyushu Island, Kagoshima Prefecture was selected. The remaining main islands, which are Hokkaido, Shikoku, and Okinawa, were not covered.

### Questionnaire

The baseline survey used a one-page questionnaire to obtain information on various factors, including tobacco smoking, alcohol drinking, dietary habits, and occupation, and, in addition, for women, their reproductive history. Questions on smoking were about the number of cigarettes smoked per day, the types of tobacco, age when starting smoking, smoking regularly or not, and, for ex-smokers, years since cessation of smoking. The amount of daily cigarette consumption was obtained from 94% of current daily cigarette smokers.^8^ An English translation of the questionnaire is given in an article written by Dr Hirayama.^1^ Table 1 summarizes questions related to lifestyles included in the questionnaire.

**Table 1.** Questions related to lifestyles included in the questionnaire used in the baseline survey

Rice/Wheat consumption	Amount/day, Frequency
Meat,	Daily/Occasionally/Rare/None/Uncertain
Fish and shellfish	Daily/Occasionally/Rare/None/Uncertain
Milk and goat milk	Daily/Occasionally/Rare/None/Uncertain
Green-yellow vegetables	Daily/Occasionally/Rare/None/Uncertain
Miso (soybean paste) soup	Daily/Occasionally/Rare/None/Uncertain
Pickles	Every meal/Daily/Occasionally/Rare/None/Uncertain
Smoking	Age started
	Daily/Occasionally/Never smoked/Uncertain
	the numbers of cigarettes smoked a day
	ex-smoker: i) the numbers of cigarettes smoked a day before cessation and ii) years after cessation
	The use of Kizami tobacco
	The use of other tobacco
Alcohol drinking	Daily/Occasionally/Rare/None/Uncertain
	Type: Sake, Shochu, Beer, Whisky, Others, Uncertain
The temperature of green tea	Very hot/Moderate/None/Uncertain

### Baseline survey

The questionnaire was distributed among all the residents at the time of the 1965 National Census, which was conducted in October, 1965. Then, they were retrieved, in most cases, by a home visit of public health nurses and midwives who were trained for a standardized method of interview. At the time of home visits, the interviewers reviewed the questionnaires filled in by the respondents, made inquiries to clarify ambiguous answers given in the questionnaire, and tried to get answers for unanswered questions. The interviewers numbered, on average, 50 in each public health district and about 1,500 in total.^8^

The proportion of respondents was 94.8% of the study area residents identified by the 1965 National Census. Prefecture-specific proportions were 99.8%, 91.4%, 91.3%, 93.8%, 95.2% and 99.2% in the prefectures of Miyagi, Aichi, Osaka, Okayama, Hyogo and Kagoshima, respectively.^8^ Table 2 shows the age and sex distributions of the respondents.^1^

**Table 2.** Study population by sex, age group and prefecture

Age, years	Prefectures					
	Miyagi	Aichi	Osaka	Hyogo	Okayama	Kagoshima
	men					
40–	7,118	7,585	7,822	6,868	6,111	6,071
50–	6,924	7,706	7,922	7,256	7,040	6,630
60–	5,605	5,849	7,191	5,850	6,802	5,537
unknown	12	18	250	24	11	59
total	19,659	21,158	23,185	19,998	19,964	18,297
	women					
40–	8,518	9,463	9,465	8,578	7,994	8,527
50–	8,072	8,962	8,923	8,549	8,530	8,264
60–	5,867	6,021	7,399	6,042	5,947	6,164
unknown	16	15	414	34	6	87
total	22,473	24,461	26,201	23,203	22,477	23,042

In 1971, an additional survey to collect information on lifestyles was conducted. Dr Hirayama described the results of this survey as follows^1^: “The information on diet was nearly as stable as that on smoking and drinking. The policy of one-time assessment of risk factors was therefore adopted for practical purposes, and information regarding risk factors obtained at the initial survey was used mostly in this study.”

### Follow-up

The cohort was followed through the end of 1982. At the beginning of each follow-up year, a migration survey was conducted through reference to the local residence registration. Those who were found to have migrated from the residential public health districts during the previous year were excluded from the mortality follow-up thereafter. During the follow-up period, 8% of the respondents migrated from the original districts. The deaths were annually ascertained through checking against vital statistics death records kept at each public health center, with the permission of the Ministry of Health and Welfare (Currently Ministry of Health, Labour and Welfare). The causes of death were coded, using the 7th Revision of the International Classification of Diseases.^8^ The information on lifestyles and other factors obtained from the baseline survey for each individual was assembled at the central office and filed systematically by residence and by date of birth. Name cards with address and key numbers were also used to help manual matching.^1^

## MAJOR PUBLICTIONS

### Second-hand smoke exposure and cancer risk

In 1981, Dr Hirayama reported an association between second-hand smoke exposure and lung cancer risk. More precisely, Hirayama Cohort Study showed that nonsmoking women married to smokers had a higher lung cancer mortality than did nonsmoking women married to nonsmokers.^9^ “Hirayama is generally credited with publishing the first evidence linking passive smoking and lung cancer” as pointed out by an article published in Bulletin of the World Health Organization in 2000.^10^ Hirayama Cohort Study is one of the most cited studies on this topic. The citation of the Hirayama paper amounted to 575 as of March, 2019 (Web of Science, Clarivate Analytics). His report was one of three spearheading epidemiological papers on the association. The other two articles were reported by Dr Dimitrios Trichopoulos and his colleagues from Greece, and Dr Lawrence Garfinkel and his colleagues from the USA.^11^^,^^12^ “While both studies showed an elevation in the point estimate of lung cancer risk associated with passive smoking, Garfinkel’s study did not reach statistical significance.”^10^ Upset by Hirayama’s study, tobacco industries tried to discredit his study results, mobilizing scientists friendly to the industry.^13^^–^^15^ As we all know, their attempts were unsuccessful. Needless to point out, Hirayama’s study was scientifically sound, as described by Sir Richard Doll, a British physician at Oxford University who did pioneering studies linking cigarette smoking and lung cancer.^16^ The evidence obtained from more than 50 additional epidemiological studies has confirmed the 1981 findings on the association between second-hand smoke exposure and lung cancer.^17^ In 1984, Dr Hirayama reported that the mortality of cancers other than lung cancer was also increased among nonsmoking women with smoking husbands.^18^ Recent meta-analysis of 4 cohort studies including Hirayama Cohort Study and 5 case-control studies estimated that the pooled relative risk of lung cancer associated with second-hand smoke exposure was 1.28 (95% confidence interval: 1.10–1.48).^19^

In the 1990s, a new cohort study (the Japan Public Health Center-based Prospective Study; JPHC study) was initiated by the National Cancer Center Japan and its collaborators. This study includes areas in Okinawa and Shikoku Islands, which were not covered by Hirayama Cohort Study. It confirmed that second-hand smoke exposure was a risk factor for lung carcinoma among Japanese women,^20^ and the association was most evident for adenocarcinoma. In the most recent and extensive pooled analyses of 18 case-control studies, which was conducted by International Lung Cancer Consortium (ILCCO), the association between second-hand smoke exposure and adenocarcinoma was statistically significant, but its magnitude was not as large as that for other histological types.^21^ Different effects of second-hand smoke exposure on different histological types of lung cancer remain to be solved.

### The associations of smoking and drinking with cancer mortality

Hirayama Cohort Study quantified the risk of cancer and other diseases in relation to tobacco smoking. “This study was large and unique in that it involved a non-Caucasian” as described by IARC monographs volume 83 on tobacco smoke and involuntary smoking.^22^ Dr Hirayama reported major findings of the cohort study at several important scientific conferences.^23^^–^^26^ The results of comprehensive analysis using Poisson regression analysis of grouped data on cancer mortality in relation to cigarette smoking were reported in 1990.^27^ Regarding alcohol drinking, Dr Hirayama summarized major findings, and also described interactions between smoking and drinking on cancer risk.^28^^,^^29^

### Dietary habits and cancer mortality

Hirayama Cohort Study revealed decreased risks of certain cancers in association with green and yellow vegetable consumption.^28^^,^^30^^,^^31^ In addition, the study found a relationship of soybean paste soup intake to gastric cancer risk.^32^ The other studies that should be mentioned here are those conducted by the collaboration with epidemiologists of Oxford University including Sir Richard Doll. One of them is on esophageal cancer mortality in relation to hot tea drinking, alcohol drinking, tobacco use and dietary habits.^33^ The other is on possible protective effect of milk, meat and fish for cerebrovascular disease mortality.^34^

## HISTORICAL IMPACT ON CANCER EPIDEMIOLOGY

The results obtained from Hirayama Cohort Study^1^ showed that the population attributable fraction in men due to daily smoking were 17.5% for all causes, 32.3% for cancer, 71.5% for lung cancer in Japan, in which the proportion of smokers among adult men was as high as 84% in 1966. The proportion declined steadily since that time; the decline sped up from 1995, and the proportion of smokers among men was under 30% in 2017 (about 8% for women). However, the health burden of smoking is still large among Japanese men as indicated by following pooled analyses of the large-scale cohort studies in Japan.^35^^,^^36^

The invaluable information obtained from Hirayama Cohort Study served as guidelines for primary prevention of cancer and other non-cancer diseases. The most important contribution of this study in the field of public health is the world-wide spread of smoking ban in indoor public venues and workplaces. Hirayama’s BMJ paper and subsequent accumulation of scientific evidence led to Article 8 of the WHO Framework Convention on Tobacco Control, which was implemented by the unrelenting efforts of people who devoted their lives to tobacco control.

It should also be noted that Hirayama Cohort Study inspired many Japanese epidemiologists. For example, the Japan Collaborative Cohort Study (JACC study) and the JPHC study can be regarded as products of such epidemiologists. The website of Japan Epidemiology Association (http://jeaweb/jp/english/activities/cohort.html/) has the list of various cohort studies conducted in Japan.

Hirayama’s work also stimulated epidemiological activities outside Japan. In India, for example, Dr Gangadharan who got an IARC scholarship with the help of Dr Hirayama and did Master of Science in Statistics at University of Pittsburgh in the 1960s, started a large-scale cohort study in Karunagappally Taluk in Kerala, south India in 1990. He was also a person inspired by Hirayama Cohort Study. The baseline survey of this cohort collected information on socioeconomic factors, lifestyles and other factors of 359,619 residents, which correspond to 93% of Karunagappally population in 1991.^37^ He and Dr Jayalekshmi, his colleague, published a number of important papers on smoking and cancer.^38^^–^^40^

In conclusion, Hirayama Cohort Study is one of the most influential cohorts in Japan and globally.
